# Cross-Sectional Area of the Rotator Cuff Muscles in MRI – Is there Evidence for a Biomechanical Balanced Shoulder?

**DOI:** 10.1371/journal.pone.0157946

**Published:** 2016-06-23

**Authors:** Samy Bouaicha, Ksenija Slankamenac, Beat K. Moor, Sina Tok, Gustav Andreisek, Tim Finkenstaedt

**Affiliations:** 1 Division of Trauma, University Hospital Zurich, Zurich, Switzerland; 2 Department of Orthopaedics, Inselspital, Bern University Hospital, Bern, Switzerland; 3 Department of Neurosurgery, Spine Center, Schulthess Clinic, Zurich, Switzerland; 4 Institute of Diagnostic and Interventional Radiology, University Hospital Zurich, University of Zurich, Zurich, Switzerland; University of Palermo, ITALY

## Abstract

**Objective:**

To provide in-vivo evidence for the common biomechanical concept of transverse and craniocaudal force couples in the shoulder that are yielded by both the rotator cuff muscles (RCM) and the deltoid and to quantitatively evaluate and correlate the cross-sectional areas (CSA) of the corresponding RCM as a surrogate marker for muscle strength using MRI.

**Materials and Methods:**

Fifty patients (mean age, 36 years; age range, 18–57 years; 41 male, 9 female) without rotator cuff tears were included in this retrospective study. Data were assessed by two readers. The CSA (mm^2^) of all rotator cuff muscles was measured on parasagittal T1-weighted FSE sequence at two different positions (at the established “y-position” and at a more medial slice in the presumably maximal CSA for each muscle, i.e., the “set position”). The CSA of the deltoid was measured on axial intermediate-weighted FSE sequences at three positions. CSA measurements were obtained using 1.5 Tesla MR-arthrographic shoulder. Pearson’s correlation for the corresponding CSA of the force couple as well as was the intraclass correlation coefficient for the inter- and intra-reader agreement was calculated.

**Results:**

The mean CSA was 770 mm^2^ (±167) and 841 mm^2^ (±191) for the supraspinatus (in the y- and set-positions, respectively) and 984 mm^2^ (±241) and 1568 mm^2^ (±338) for the infraspinatus. The mean CSA was 446 mm^2^ (±129) and 438 mm^2^ (±128) for the teres minor (in the y- and set-positions, respectively) and 1953 mm^2^ (±553) and 2343 mm^2^ (±587) for the subscapularis. The three measurements of the deltoid revealed a CSA of 3063 mm^2^ (±839) for the upper edge, 3829 mm^2^ (±836) for the lower edge and 4069 mm^2^ (±937) for the middle of the glenoid. At the set position Pearson’s correlation of the transverse force couple (subscapularis/infraspinatus) showed a moderate positive correlation of r = 0.583 (p<0.0001) and a strong correlation when the CSA of the teres minor was added to the infraspinatus CSA (r = 0.665, p = 0.0008) and a strong positive correlation of the craniocaudal force couple (supraspinatus/deltoid) that ranged from r = 0.565–0.698 (p<0.0001). Inter-reader agreement (ranged from 0.841 to 0.997, p = 0.0007) and intra-reader agreement were excellent (ranged from 0.863 to 0.999, p = 0.0006).

**Conclusion:**

The significant correlation of the CSA of the RCM that form the transverse (subscapularis/infraspinatus-teres minor) and craniocaudal (supraspinatus/deltoid) force couple measured by MR-arthrography supports the biomechanical concept of a dynamically balanced shoulder in patients with an intact rotator cuff.

## Introduction

The intricate biomechanical concept of glenohumeral stabilization is based on static and dynamic stabilization and involves two important stabilizing mechanisms, concavity compression and glenohumeral balance, as proposed in 1993 by Lippitt et al. [1]. Static soft-tissue stabilization by the joint capsule and glenohumeral ligaments is mainly provided in the end-range joint position. Because of the unconstrained geometry of the glenohumeral joint, the four rotator cuff muscles serve as the main dynamic stabilizers of the humeral head [1–3], with contributing roles of other muscles including deltoid, latissimus dorsi, teres major, biceps and triceps depending on the joint position. The antagonist subscapularis and infraspinatus/teres minor muscles build a force couple that centers the humeral head in an anteroposterior direction [4, 5]. The supraspinatus works–depending on the joint position—primarily as the counteracting force to the cranial pull of the deltoid muscle [6, 7]. To ensure the stabilization of the humeral head with a high degree of range of motion, balanced forces of the contributing muscles are mandatory. Any asymmetric tonus of the agonists results in altered glenohumeral shear forces that might lead to displacing of the proximal humerus from its center of rotation in the glenoid cavity and thus might facilitate joint attrition and cause subluxation of the proximal humerus as seen in shoulders with major defects of the rotator cuff [5, 7]. Knowledge of a biomechanical balanced shoulder is crucial for surgical therapy planning, e.g. latissimus dorsi transfer, shoulder prosthesis implantation or treating of torn rotator cuff tendons.

Many studies have shown that torn rotator cuff tendons are associated with muscle atrophy and fatty infiltration, which indicate a loss of muscle strength, function, and altered joint reaction forces [8, 9]. Also muscle size and joint strength are known to change throughout the adult lifespan. Muscle volume decreases in older adults particularly in the shoulder, however, the linear relationship between muscle volume and joint-moment generating capacity is maintained in older adults [10–12]. The strength of a muscle can be calculated from its muscle volume [13] and cross-sectional area (CSA), and are therefore a useful surrogate marker for the applied tension forces in the biomechanical analysis of joint reaction forces [14, 15]. Computed tomography (CT) and magnetic resonance imaging (MRI) are known for providing reliable measurements of rotator cuff muscle sizes, particularly the CSA [16, 17]. This current study initially enables an in-vivo assessment of the muscle CSA and provides an examination of the association between the muscles in the transverse and craniocaudal force couples in the shoulder.

Based on this knowledge, we hypothesized that the CSA of the corresponding force couple muscles of the rotator cuff and the deltoid measured on MRI correlate with each other and reflect an in-vivo proof of the well-known, in-vitro explored concept of a balanced shoulder in adult patients with healthy rotator cuffs.

## Materials and Methods

### Study Population

Institutional review board approval was obtained by the local ethics committee "Cantonal Ethics Committee Zurich" and conducted in compliance with the Declaration of Helsinki. Written informed patient consent was waived by our local ethics committee due to the retrospective nature of the study. The patient records/information were anonymized and de-identified prior to analysis.

**Fig 1:**
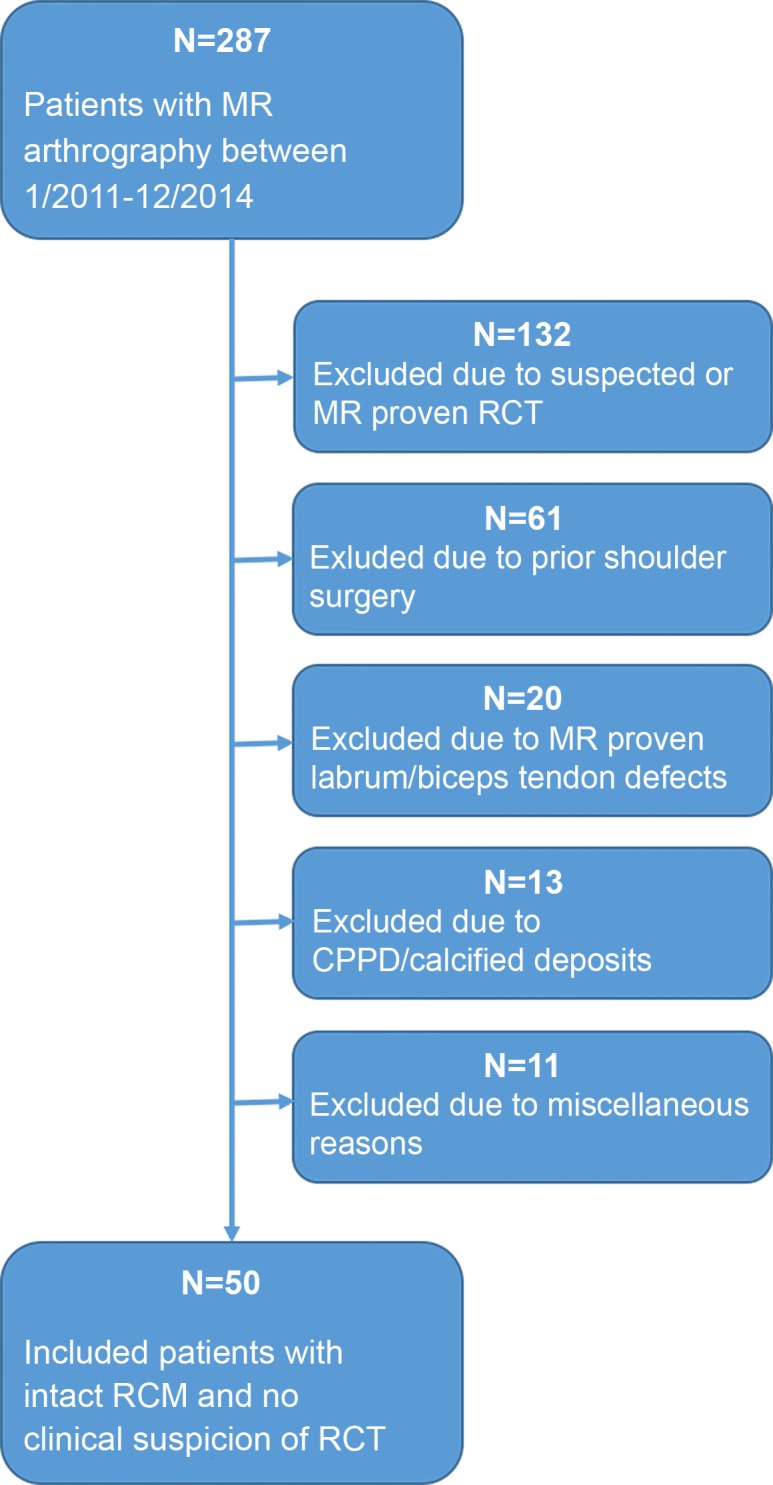
Flow chart of subject inclusion. RCT = rotator cuff tear, CPPD = Calcium pyrophosphate dihydrate deposition disease, RCM = rotator cuff muscles.

Two hundred eighty-seven patients who were referred to magnetic resonance arthrography (MR arthrography) between January 2011 and December 2014 were screened for study inclusion (**Fig 1**). Fifty patients (9 female, 41 male; female age range, 27–54 years; mean age, 43 ± 10 years; male age range, 18–57 years; mean age, 34 ± 13 years; mean age of all patients: 36 ± 13 years) of the shoulder (right: 26 (23/26 dominant side); left: 24 (20/24 dominant side) were included in this retrospective study. All fifty MR arthrographies arise from fifty different patients.

MR arthrography was clinically indicated in all patients and included suspected lesions of both the labrum and the biceps tendon. Inclusion criteria were no significant findings according to MR arthrography that may alter glenohumeral stabilization, notably an intact rotator cuff diagnosed on MR arthrography and no clinical suspicion of rotator cuff pathologies. Exclusion criteria were prior shoulder surgery, history or clinical suspicion of rotator cuff pathology, history of crystal deposition diseases (i.e., CPPD) and when calcifications at the shoulder were visible on the fluoroscopic image from the arthrography or conventional radiography, as were any muscle disease, suspected or proven adhesive capsulitis, major deformity of the shoulder girdle, history of asymmetric shoulder use or muscle training or neurologic disease that might influence the peripheral muscles. Also patients with significant defects of the labrum and/or the biceps tendon proven by the MR arthrography were excluded.

### Imaging

MR arthrography included intraarticular injection of the contrast agent under fluoroscopic guidance as well as subsequent MR imaging using a 1.5-T scanner (Signa Excite HD, GE Healthcare, Wauwatosa, WI, USA) and a dedicated shoulder array coil (8-channel shoulder array, GE Healthcare, Wauwatosa, WI, USA). The MR protocol was the established protocol for shoulder MR arthrography at our hospital. It consisted of a parasagittal T1-weighted fast spin echo (FSE) (repetition time/echo time (TR/TE), 595/8.3 ms; slice thickness, 3 mm; field of view (FOV), 140 mm; matrix, 416 x 256), a fat-suppressed parasagittal T2-weighted FSE (TR/TE, 4400/92 ms; slice thickness, 3 mm; FOV, 140 mm; matrix, 256 x 224), a paracoronal fat-suppressed T1-weighted FSE (TR/TE, 494/13 ms; slice thickness, 3 mm; FOV, 140 mm, matrix, 256 x 224), a paracoronal intermediate-weighted FSE (TR/TE, 2261/37 ms; slice thickness, 3 mm; FOV, 140 mm; matrix, 352 x 256), an axial intermediate-weighted FSE (TR/TE, 3229/36 ms; slice thickness, 3 mm; FOV, 140 mm; matrix, 256 x 224) and an axial three-dimensional gradient echo (TR/TE, 9.8/4.8 ms; slice thickness, 2 mm; FOV, 180 mm; matrix, 512 x 320). The scan time for each patient was approximately 24 minutes.

### Image Analysis

Measurements of the cross-sectional areas were performed by a radiologist (*R1*, *initials—blinded for review*) with four years of experience in musculoskeletal imaging using dedicated software (Myrian; Intrasense, Paris, France). In addition, intra-reader and inter-reader agreement was systematically assessed. In order to determine the intra-reader agreement, image analysis of 50% of the randomly selected cases (n = 25) was repeated by R1 (after a time interval of four weeks to avoid recall bias). In order to determine the inter-reader agreement, a second reader (*R2*, *initials—blinded for review*) with four years of experience in musculoskeletal imaging performed an image analysis of the same randomly selected 50% of the cases (n = 25). Images were shown randomly and both readers were always blinded to the name and details of the patients. R1 and R2 did not converse with one another before reviewing the scans to agree upon a specific approach.

**Fig 2:**
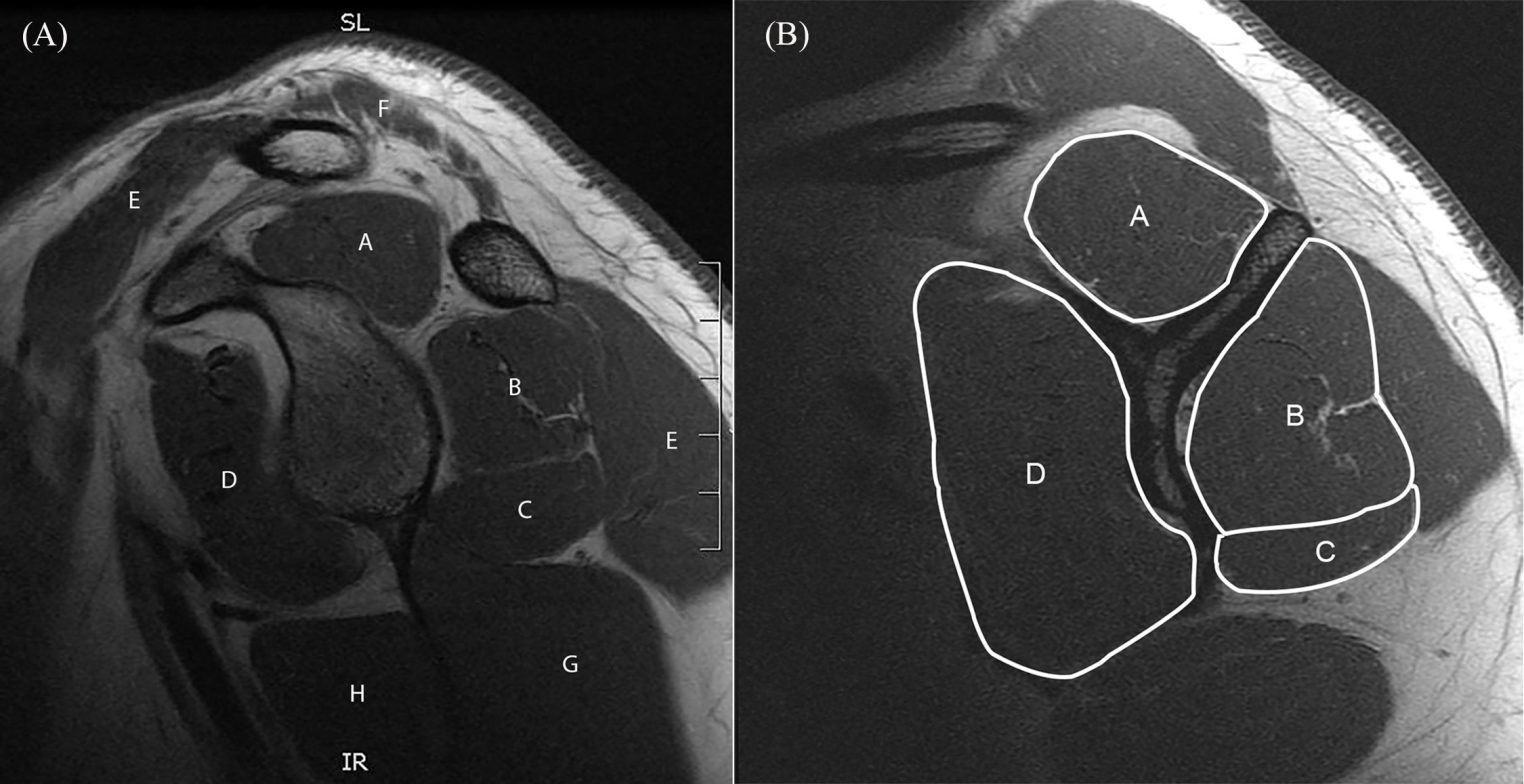
Anatomy of shoulder girdle and measurement example for “y-position”. Left panel shows a sagittal T1-weighted MR-arthrography image of the right shoulder of a thirty-five-year-old male, and depicts the anatomy of the shoulder girdle muscles on the level of the glenoid cavity: supraspinatus (A), infraspinatus (B), teres minor (C), subscapularis (D), deltoid (E), trapezius (F), triceps brachii (G) and teres major (H). Right panel illustrates one of the two positions where the cross-sectional area (CSA) of the rotator cuff muscles was measured: the most lateral slice, where the corpus scapulae and the spina scapulae had a Y-shaped appearance. CSA in this patient measured supraspinatus (793 mm^2^, A), infraspinatus (969 mm^2^, B), teres minor (515 mm^2^, C) and subscapularis (1761 mm^2^, D).

**Fig 3:**
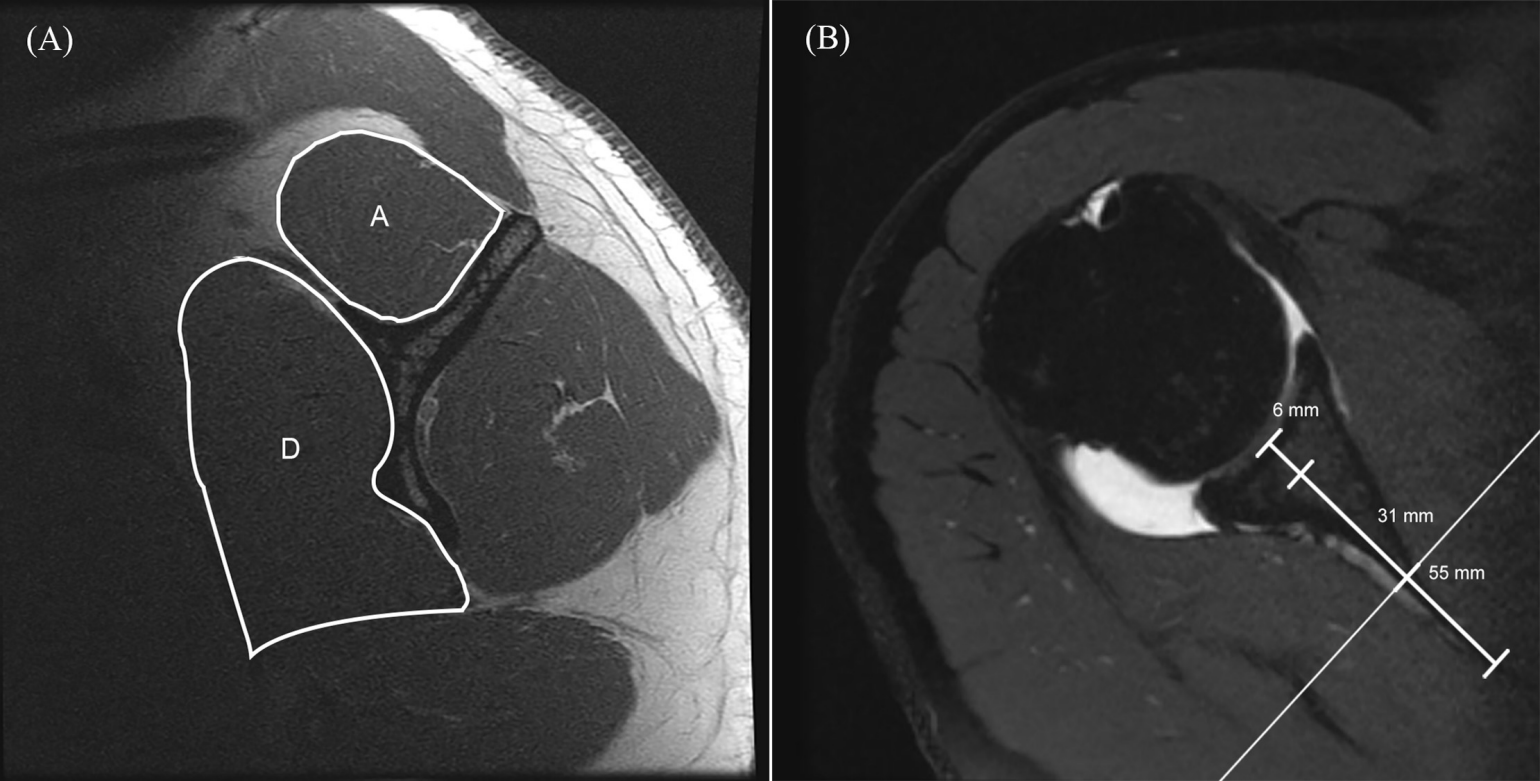
Measurement example for “set position”. According to Yanagisawa et al. [19] the cross-sectional area (CSA) measurement of the rotator cuff muscles was also performed at more medial slices. Left panel shows a sagittal T1-weighted MR-arthrography image of the same patient (see Figs 2 and 4) and highlights the CSA measurement of the supraspinatus (955 mm^2^, A) and subscapularis (2306 mm^2^, D) 31 mm medial to the center of the glenoid cavity. The right panel, which shows an axial PD-weighted image, indicates the suggested measurement levels for males as the medial distance from the glenoid cavity base: 6 mm for the CSA measurement of the teres minor, 31 mm for supraspinatus and subscapularis (see reference line) and 55 mm for infraspinatus.

For quantitative CSA rotator cuff muscle measurement, the parasagittal T1-weighted FSE sequences were used with the help of axial intermediate-weighted FSE sequences to localize the distance from the center of the glenoid cavity. The CSA (mm^2^) of all rotator cuff muscles was measured at two different positions on the parasagittal images. First, at the most lateral slice where the corpus scapulae and the spina scapulae had a Y-shaped appearance [18] and the spongiosa of the spina scapulae was observed (referred to as the “y-position”, **Fig 2**). Second, at a more medial slice, according to the method described by Yanagisawa et al.[19] (referred to as the “set position”). The CSA of the supraspinatus muscle was measured 31 mm in males and 27 mm in females medial to the center of the glenoid cavity. The infraspinatus muscle was measured 55 mm in males and 51 mm in females medial to the center of the glenoid cavity. The teres minor muscle was measured 6 mm in males and 5 mm in females medial to the center of the glenoid cavity. The subscapularis muscle was measured 31 mm in males and 38 mm in females medial to the center of the glenoid cavity (**Fig 3**). Due to the similar muscle function of the infraspinatus and the teres minor their mutual CSA was calculated and used for the correlations at both measurement positions, the y-position and set position.

**Fig 4:**
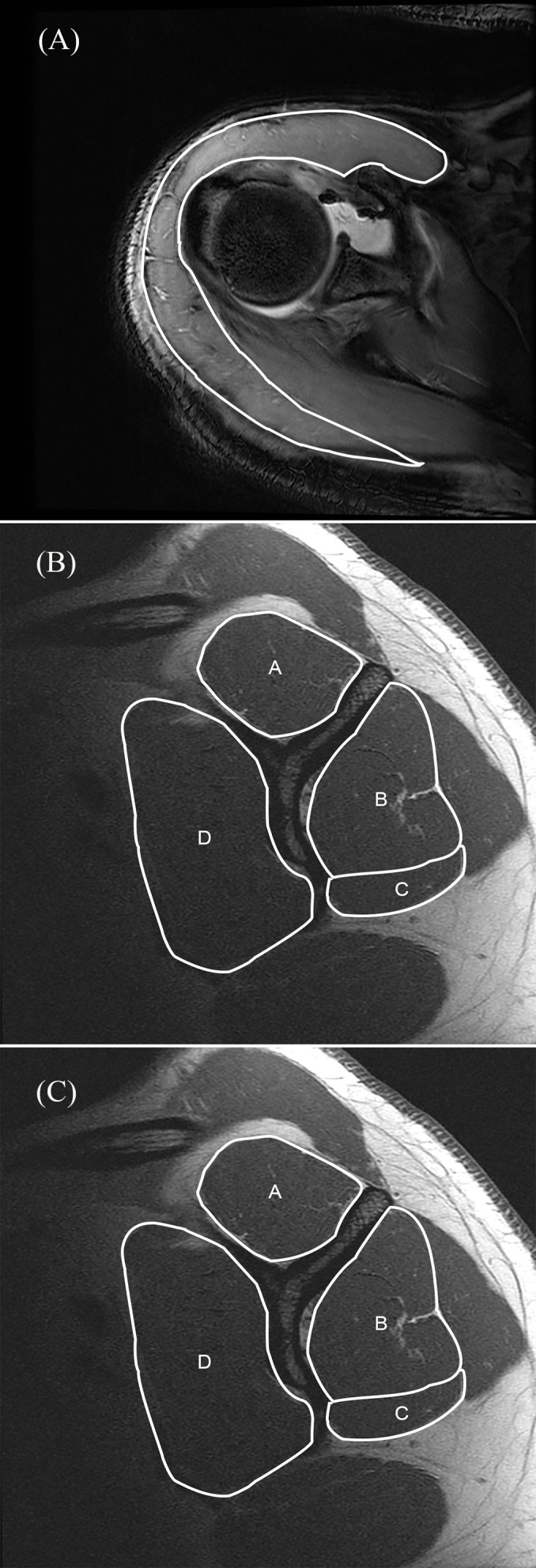
Measurement example deltoid. For the cross-sectional area (CSA) measurement of the deltoid muscle no reference in the literature existed. Thus, three measurement levels have been defined on axial three-dimensional gradient echo MR-arthrography sequences: CSA was measured at the upper- (2816 mm^2^, top panel) and lower edge (4633 mm^2^, bottom panel) and the middle of the glenoid (4002 mm^2^, middle panel). Measurements were performed on the images of the same patient (see Figs 2 and 3).

Additionally, the CSA of the deltoid muscle was measured on the axial intermediate-weighted FSE sequences at three positions: the upper and lower edges and the middle of the glenoid (**Fig 4**). The measurement at the middle position was performed exactly at the mid-glenoid level according to the method described earlier by Meyer et al. [20] and included all segments of the deltoid deriving from the clavicle, acromion and scapular spine. Fatty infiltration of the rotator cuff muscles was not assessed.

### Statistical Analysis

We expressed the distribution of variables using means and standard deviation (SD) for normally distributed data and medians and interquartile ranges for non-normally distributed data. After confirming normality of the data with the Kolmogorow-Smirnov test Pearson’s correlation coefficient was used to test for correlations of the CSA of the rotator cuff muscles as well as the deltoid muscle. According to Evans [21] Pearson correlation coefficient values of 0.4–0.59 indicated moderate agreement, values of 0.6–0.79 strong agreement and values greater than 0.8 excellent agreement. The intraclass correlation coefficient (ICC) was computed to assess the inter- and intra-reader agreement. The ICC values have been interpreted according to Cicchetti [22]: Values of less than 0.40 indicate poor; less than 0.59, fair; less than 0.74, good; and greater than 0.75, excellent agreement.

Using the technique of Li and Ji to correct multiple comparisons [23], six effective variables were found that required correction. Consequently, a p-value of <0.0033 was considered statistically significant. All statistical calculations were performed using commercially available software (IBM SPSS Statistics, Version 21.0.: IBM Corp. Armonk, NY, USA).

## Results

### Inter- and intra-reader agreement

Intraclass correlation coefficient (ICC) for the inter-reader agreement (ranged from 0.841 to 0.997, p = 0.0007) as well as intra-reader agreement was excellent (ranged from 0.863 to 0.999, p = 0.0006).

### CSA measurement

**Fig 5:**
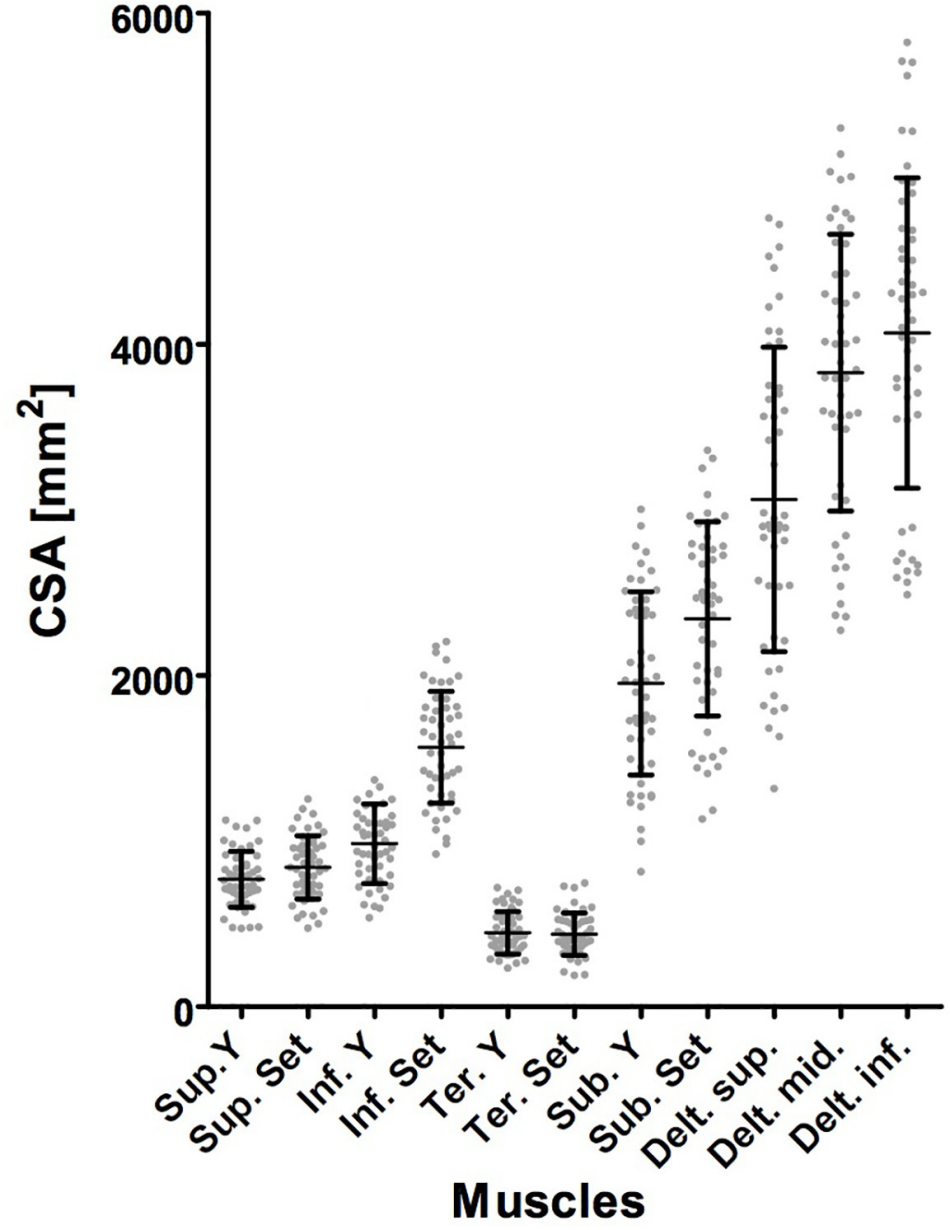
Distribution of cross-sectional area measurements. The scatterplot displays the distribution of the measured cross-sectional area (CSA) for each muscle and the respective standard deviation indicated by the margins of the bar. The y-axis describes the measured CSA in the unit mm^2^, whereas the x-axis describes the various measured muscles.

Descriptive statistics showed a mean CSA of the supraspinatus muscle of 770 mm^2^ (standard deviation (±) 167 mm^2^) at the y-position and 841 mm^2^ (± 191 mm^2^) at the set position; for the infraspinatus muscle, it was 984 mm^2^ (± 241 mm^2^) at the y-position and 1568 mm^2^ (± 338 mm^2^) at the set position. The mean values for the teres minor muscle were 446 mm^2^ (± 129 mm^2^) at the y-position and 438 mm^2^ (± 128 mm^2^) at the set position. The mean CSA of the subscapularis was 1953 mm^2^ (± 553 mm^2^) at the y-position and 2343 mm^2^ (± 587) at the set position. The three measurements of the deltoid revealed a mean area of 3063 mm^2^ (± 839 mm^2^, range 1317–4763 mm^2^) for the upper edge, 3829 mm^2^ (± 836 mm^2^, range 2247–5306 mm^2^) for the lower edge and 4069 mm^2^ (± 937 mm^2^, range 2489–5825 mm^2^) for the middle of the glenoid. Results are summarized as scatter plots in **Fig 5**. Our measurements were similar to those of Yanagisawa et al. [19] and Meyer et al. [20] (**Table 1**).

**Table 1:** Comparison between CSA measurements in the literature. Table shows our results for the cross-sectional area (CSA) measurements at the set position in comparison to the measurements for rotator cuff muscles by Yanagisawa et al. [19], who suggested the set position to be most appropriate for maximal CSA assessment. Yanagisawa et al. divided the results into the groups males (♂) and females (♀). Additionally, the CSA measurements of the deltoid by Meyer et al. [20] were listed. All values are mean values with the unit mm^2^; “±” represents the standard deviation.

	Current study	Yanagisawa et al.	Meyer et al.
**Supraspinatus**	841 ± 191	♂ 809 ± 100	-
		♀ 541 ± 86	
**Infraspinatus**	1568 ± 338	♂ 1584 ± 183	-
		♀ 1060 ± 173	
**Teres minor**	438 ± 128	♂ 360 ± 121	-
		♀ 294 ± 66	
**Subscapularis**	2343 ± 587	♂ 2428 ± 370	-
		♀ 1506 ± 213	
**Deltoid,**	4069 ± 937	-	3896 ±1317
	(range 2489–5825)		
			(range 1566–7484)
**mid-glenoid level**			

### CSA measurement correlation

Pearson’s correlation of the transverse force couple (subscapularis and infraspinatus) showed a moderate positive correlation of r = 0.583 (p<0.0001) at the set measurement position and a strong correlation when the CSA of the teres minor was added to the infraspinatus CSA (subscapularis/infraspinatus-teres minor, r = 0.665, p = 0.0008). At the y-position there was a moderate positive correlation of r = 0.428 (p = 0.0006) and a slightly higher correlation when the CSA of the teres minor was added to the infraspinatus CSA (r = 0.526, p = 0.0007) (**Table 2**).

**Table 2:** Correlation of muscle CSA measurements for transverse force couple. Table summarizes the results of the correlation of the cross-sectional area (CSA) measurements between the transverse force couple (Inf. = M. infraspinatus, Ter. = M. teres minor; Sub. = M. subscapularis). CSA measurements of the rotator cuff muscles were performed at two different positions: the “y-position” and the “set position”.

	Inf. Set	Ter. Y	Ter. Set	Sub. Y	Sub. Set	Inf. + Ter. Y	Inf. + Ter. Set
**Inf. Y**	R = 0.591	R = 0.322	R = 0.401	R = 0.428**	R = 0.553**	R = 0.918	R = 0.614
	p<0.0001	p = 0.0225	p = 0.0039	p = 0.0006	p<0.0001	p<0.0001	p<0.0001
**Inf. Set**	x	R = 0.426	R = 0.422	R = 0.497**	R = 0.583**	R = 0.641	R = 0.959
		p = 0.0021	p = 0.0029	p<0.0002	p<0.0001	p<0.0001	p<0.0001
**Ter. Y**	x	x	R = 0.678	R = 0.456**	R = 0.549**	R = 0.671	R = 0.564
			p<0.0001	p = 0.009	p<0.0001	p<0.0001	p<0.0001
**Ter. Set**	x	x	x	R = 0.497**	R = 0.585**	R = 0.598	R = 0.661
				p = 0.0002	p<0.0001	p<0.0001	p<0.0001
**Sub. Y**	x	x	x	x	R = 0.674	R = 0.526**	R = 0.566**
					p<0.0001	p = 0.0007	p<0.0001
**Sub. Set**	x	x	x	x	x	R = 0.663***	R = 0.665***
						p<0.0001	p = 0.0008

** indicate moderate

*** strong agreement values of the Pearson correlation coefficient

A moderate/strong positive correlation of the craniocaudal force couple (supraspinatus and deltoid), which ranged from r = 0.565–0.698 (p<0.0001) at the set position and 0.565–0.639 (p<0.0001) at the y-position, with the highest correlation between the supraspinatus and the middle deltoid at the set position (r = 0.698, p<0.0001) and the y-position (r = 0.639, p<0.0001), was found (**Table 3**).

**Table 3:** Correlation of muscle CSA measurements for craniocaudal force couple. Table summarizes the results of the correlation of the cross-sectional area (CSA) measurements between the craniocaudal force couple (Sup. = M. supraspinatus and the deltoid (Delt. sup. = M. deltoideus, at the upper edge of glenoid; Delt. mid. = M. deltoideus, at the middle of the glenoid; Delt. inf. = M. deltoideus, at the lower edge of glenoid). CSA measurements of the rotator cuff muscles were performed at two different positions: the “y-position” and the “set position”.

	Sup. Set	Delt. sup.	Delt. mid.	Delt. inf.
**Sup. Y**	R = 0.950	R = 0.565**	R = 0.639***	R = 0.594**
	p<0.0001	p<0.0001	p<0.0001	p<0.0001
**Sup. Set**	x	R = 0.565**	R = 0.698***	R = 0.638***
		p<0.0001	p<0.0001	p<0.0001
**Delt. sup.**	x	x	R = 0.831	R = 0.699
			p<0.0001	p<0.0001
**Delt. mid.**	x	x	x	R = 0.922
				p<0.0001

** indicate moderate

*** strong agreement values of the Pearson correlation coefficient

In contrast to the moderate to strong correlations for the force couples, other non-corresponding force couples showed predominantly weaker values. The comparison of the infraspinatus and the deltoid revealed also a comparable positive correlation for the y-position (ranged from r = 0.433–0.649, p**≤**0.0017) and set position (ranged from r = 0.448–0.585, p≤0.0011) as well as the comparison of the subscapularis and the deltoid (ranged from r = 0.422–0.539, p≤0.0023) at the set position.

## Discussion

The concept of the transverse and craniocaudal force couples in the shoulder was investigated in several biomechanical studies [4–7]. Since static soft-tissue stabilization by the joint capsule and glenohumeral ligaments is mainly provided in end-range joint positions, dynamic neuro-muscular control is crucial for all mid- and low-range movements to keep the proximal humerus centered to the glenoid cavity. Therefore, the understanding of muscular physiology and anatomy around the glenohumeral joint is mandatory and can serve as a decision-making aid for conservative and surgical therapeutic treatment planning. Thus, knowledge about the performance of the anteroposterior and craniocaudal force couples is of clinical significance, e.g. for determination whether anatomical total shoulder replacement or reverse total shoulder replacement should be applied. If the strength of the supraspinatus is insufficient and consequently the deltoid pulls the humerus upwards, reverse total shoulder replacement is favored over anatomical shoulder replacement to prevent unfavorable clinical outcomes [24, 25]. Additionally, preoperative knowledge about deltoid strength is important as reverse total shoulder replacement results in medialization and distalization of the center of rotation of the shoulder joint and therefore increases the deltoid muscle moment arm. Hence, it is vital to anticipate whether the deltoid will be able to perform as the primary contributor for shoulder movement postoperatively [26, 27]. Furthermore, preoperative anticipation of subscapularis performance is essential for latissimus dorsi transfer planning, because its insufficiency constitutes a contraindication due to the potential anterior subluxation of the humeral head [28, 29]. Clinical assessment of the strength and condition of the antagonist rotator cuff muscles and deltoid can be difficult and therefore CSA assessment of the participating muscles as an indicator for muscle force can aid clinical work-up.

A muscle's cross-sectional area is a well-established surrogate marker for the force produced by the muscle. The force is directed along the muscular line of action (which can change with joint angle and attachment location) and determines the moment arm about the glenohumeral joint [20]. The measurement of the CSA might be a little less appropriate for the evaluation of muscle force compared to measurement of muscle volume [13, 30, 31], but represents not only an easier and less time consuming measuring technique, but also is more suitable for clinical routine, because most MRI examinations do not cover the entire rotator cuff muscles on the medial side to save scanning time.

The mean CSA findings in the present study for the rotator cuff muscles were similar to the data reported by Yanagisawa et al. [19]. In their study CSA measurements on MRI were steadily performed on each whole muscle and the maximum CSA for each muscle was found to be on a more medial slice compared to the established y-position [18]. The maximum CSA values were determined to be similar in athletes as well as nonathletes of both sexes. Thus we have included both measurement positions, the y- and set-position, in our study for CSA assessment of the four rotator cuff muscles. Previously there is only the study of Meyer et al. that proposed a CSA measurement position for the deltoid [20], namely the mid-glenoid level. We developed an advanced measurement technique with two additional measuring positions to ascertain the deltoid cross-sectional area at three defined levels. Our measurement results for the mid-glenoid level are comparable to those described by Meyer et al. (**Table 1**).

With significant positive correlations of the CSAs for the transverse (moderate, respectively strong when the CSA of the functional similar teres minor was added to the infraspinatus CSA) and craniocaudal (strong) force couples on MRI, we initially showed in-vivo evidence for a muscular-balanced shoulder in healthy adults. These findings are in concordance with a biomechanical precondition for a stable glenohumeral joint. The highest correlations for the deltoid muscles with the CSA of the corresponding supraspinatus were found in the mid-glenoid level in the values for both the y- and the set-position. This result may reflect our mid-glenoid measurement position that features the greatest CSA to be most representative for the deltoid force.

Other non-corresponding force couples showed predominantly weaker values except for the positive correlations for the infraspinatus/teres minor and the deltoid as well as the subscapularis and the deltoid. These findings might be explained by the diverse functions of the deltoid: the anterior fibers are not only involved in shoulder abduction when the shoulder is externally rotated, but also facilitate internal rotation of the humeral head and thus have synergetic effects with the subscapularis. The posterior fibers are strongly involved in transverse extension and external rotation of the humeral head and thus have synergetic effects with the infraspinatus/teres minor. Furthermore, biomechanical studies have shown that the supraspinatus and deltoid work synergistically or antagonistically, depending on different shoulder positions; inherently, an interpretation of our static findings with the arm in a standardized adducted position in the MR scanner does not reflect the different arm positions and muscle functions.

Our study has some limitations: First, despite strict inclusion and exclusion criteria there might be other shoulder pathologies with concomitant inactivity or immobilization that may influence the CSA of the rotator cuff muscles and deltoid. Second, the CSA of the force couples may be influenced as well by selective physical training and their association might be age-dependent, although we did not find marked differences after covariance analysis for the variable age. Third, the slice thickness of the parasagittal T1-weighted FSE sequence used for the measurements were 3 mm as it is standard in the clinical setting and thus in the most unfavorable case might lead to a misplacement of the measurement position of maximal 1.5 mm. Finally, CSA measurements at the y- and set-position may not depict the maximal CSA due to different anthropometry.

## Conclusion

In conclusion, based on our MRI measurements the significant correlations of the cross-sectional areas of the rotator cuff muscles that form the transverse (subscapularis/infraspinatus-teres minor) and craniocaudal (supraspinatus/deltoid) force couple support the biomechanical concept of a dynamically balanced shoulder in adult patients with an intact rotator cuff. Knowledge about the muscle forces of the rotator cuff muscles is the basis for further research and might impact the therapeutic decision making of orthopedic surgeons facing patients with rotator cuff tears or candidates for shoulder prosthesis implantation.

## References

[1] Lippitt S, Matsen F. Mechanisms of glenohumeral joint stability. Clin Orthop Relat Res. 1993;(291):20–8. 8504601

[2] Favre P, Senteler M, Hipp J, Scherrer S, Gerber C, Snedeker JG. An integrated model of active glenohumeral stability. J Biomech. 2012;45(13):2248–55. 10.1016/j.jbiomech.2012.06.010 22818663

[3] McMahon PJ, Lee TQ. Muscles may contribute to shoulder dislocation and stability. Clin Orthop Relat Res. 2002;(403 Suppl):S18–25. 10.1097/00003086-200210001-00003 12394449

[4] Ackland DC, Pandy MG. Lines of action and stabilizing potential of the shoulder musculature. J Anat. 2009;215(2):184–97. 10.1111/j.1469-7580.2009.01090.x 19490400 PMC2740966

[5] Thompson WO, Debski RE, Boardman NDv 3rd Taskiran E, Warner JJ, Fu FH, et al A biomechanical analysis of rotator cuff deficiency in a cadaveric model. Am J Sports Med. 1996;24(3):286–92. 8734877 10.1177/036354659602400307

[6] Sharkey NA, Marder RA. The rotator cuff opposes superior translation of the humeral head. Am J Sports Med. 1995;23(3):270–5. 7661251 10.1177/036354659502300303

[7] Hansen ML, Otis JC, Johnson JS, Cordasco FA, Craig EV, Warren RF. Biomechanics of massive rotator cuff tears: implications for treatment. J Bone Joint Surg Am. 2008;90(2):316–25. 10.2106/JBJS.F.00880 18245591

[8] Meyer DC, Gerber C, Von Rechenberg B, Wirth SH, Farshad M. Amplitude and strength of muscle contraction are reduced in experimental tears of the rotator cuff. Am J Sports Med. 2011;39(7):1456–61. 10.1177/0363546510396305 21350068

[9] Gerber C, Schneeberger AG, Hoppeler H, Meyer DC. Correlation of atrophy and fatty infiltration on strength and integrity of rotator cuff repairs: a study in thirteen patients. J Shoulder Elbow Surg. 2007;16(6):691–6. 17931904 10.1016/j.jse.2007.02.122

[10] Saul KR, Vidt ME, Gold GE, Murray WM. Upper Limb Strength and Muscle Volume in Healthy Middle-Aged Adults. Journal of applied biomechanics. 2015;31(6):484–91. 10.1123/jab.2014-0177 26155870

[11] Vidt ME, Daly M, Miller ME, Davis CC, Marsh AP, Saul KR. Characterizing upper limb muscle volume and strength in older adults: a comparison with young adults. J Biomech. 2012;45(2):334–41. 10.1016/j.jbiomech.2011.10.007 22047782 PMC3246044

[12] Holzbaur KR, Murray WM, Gold GE, Delp SL. Upper limb muscle volumes in adult subjects. J Biomech. 2007;40(4):742–9. 17241636 10.1016/j.jbiomech.2006.11.011

[13] Akagi R, Takai Y, Ohta M, Kanehisa H, Kawakami Y, Fukunaga T. Muscle volume compared to cross-sectional area is more appropriate for evaluating muscle strength in young and elderly individuals. Age and ageing. 2009;38(5):564–9. 10.1093/ageing/afp122 19596739

[14] Bamman MM, Newcomer BR, Larson-Meyer DE, Weinsier RL, Hunter GR. Evaluation of the strength-size relationship in vivo using various muscle size indices. Med Sci Sports Exerc. 2000;32(7):1307–13. 10912898 10.1097/00005768-200007000-00019

[15] Masuda K, Kikuhara N, Takahashi H, Yamanaka K. The relationship between muscle cross-sectional area and strength in various isokinetic movements among soccer players. J Sports Sci. 2003;21(10):851–8. 14620028 10.1080/0264041031000102042

[16] Fuchs B, Weishaupt D, Zanetti M, Hodler J, Gerber C. Fatty degeneration of the muscles of the rotator cuff: assessment by computed tomography versus magnetic resonance imaging. J Shoulder Elbow Surg. 1999;8(6):599–605. 10633896 10.1016/s1058-2746(99)90097-6

[17] Spencer EE Jr., Dunn WR, Wright RW, Wolf BR, Spindler KP, McCarty E, et al Interobserver agreement in the classification of rotator cuff tears using magnetic resonance imaging. Am J Sports Med. 2008;36(1):99–103. 17932406 10.1177/0363546507307504

[18] Zanetti M, Gerber C, Hodler J. Quantitative assessment of the muscles of the rotator cuff with magnetic resonance imaging. Investigative radiology. 1998;33(3):163–70. 9525755 10.1097/00004424-199803000-00006

[19] Yanagisawa O, Dohi M, Okuwaki T, Tawara N, Niitsu M, Takahashi H. Appropriate slice location to assess maximal cross-sectional area of individual rotator cuff muscles in normal adults and athletes. Magn Reson Med Sci. 2009;8(2):65–71. 19571498 10.2463/mrms.8.65

[20] Meyer DC, Rahm S, Farshad M, Lajtai G, Wieser K. Deltoid muscle shape analysis with magnetic resonance imaging in patients with chronic rotator cuff tears. BMC musculoskeletal disorders. 2013;14:247 10.1186/1471-2474-14-247 23957805 PMC3751864

[21] Evans JD. Straightforward statistics for the behavioral sciences Pacific Grove: Brooks/Cole Pub Co 1996:591–2.

[22] DV C. Guidelines, criteria, and rules of thumb for evaluating normed and standardized assessment instruments in psychology. Psychol Assess. 1994;(6):284–90.

[23] Li J, Ji L. Adjusting multiple testing in multilocus analyses using the eigenvalues of a correlation matrix. Heredity. 2005;95(3):221–7. 16077740 10.1038/sj.hdy.6800717

[24] Flurin PH, Marczuk Y, Janout M, Wright TW, Zuckerman J, Roche CP. Comparison of outcomes using anatomic and reverse total shoulder arthroplasty. Bull Hosp Jt Dis (2013). 2013;71 Suppl 2:101–7. 24328590

[25] Gerber C, Pennington SD, Nyffeler RW. Reverse total shoulder arthroplasty. J Am Acad Orthop Surg. 2009;17(5):284–95. 19411640 10.5435/00124635-200905000-00003

[26] Farshad M, Gerber C. Reverse total shoulder arthroplasty-from the most to the least common complication. Int Orthop. 2010;34(8):1075–82. 10.1007/s00264-010-1125-2 20865260 PMC2989053

[27] Guery J, Favard L, Sirveaux F, Oudet D, Mole D, Walch G. Reverse total shoulder arthroplasty. Survivorship analysis of eighty replacements followed for five to ten years. J Bone Joint Surg Am. 2006;88(8):1742–7. 16882896 10.2106/JBJS.E.00851

[28] Gerber C. Latissimus dorsi transfer for the treatment of irreparable tears of the rotator cuff. Clin Orthop Relat Res. 1992;(275):152–60. 1735206

[29] Werner CM, Zingg PO, Lie D, Jacob HA, Gerber C. The biomechanical role of the subscapularis in latissimus dorsi transfer for the treatment of irreparable rotator cuff tears. J Shoulder Elbow Surg. 2006;15(6):736–42. 17126245 10.1016/j.jse.2005.11.002

[30] De Ste Croix M, Deighan M, Armstrong N. Assessment and interpretation of isokinetic muscle strength during growth and maturation. Sports medicine. 2003;33(10):727–43. 12895130 10.2165/00007256-200333100-00002

[31] Lehtinen JT, Tingart MJ, Apreleva M, Zurakowski D, Palmer W, Warner JJ. Practical assessment of rotator cuff muscle volumes using shoulder MRI. Acta orthopaedica Scandinavica. 2003;74(6):722–9. 14763706 10.1080/00016470310018270

